# Effector-dependent neural representations of perceptual decisions independent of motor actions and sensory modalities

**DOI:** 10.1162/IMAG.a.11

**Published:** 2025-05-22

**Authors:** Marlon F. Esmeyer, Timo T. Schmidt, Felix Blankenburg

**Affiliations:** Neurocomputation and Neuroimaging Unit, Department of Education and Psychology, Freie Universität Berlin, Berlin, Germany; Berlin School of Mind and Brain, Humboldt-Universität zu Berlin, Berlin, Germany

**Keywords:** multisensory decision-making, effector-dependent, delayed match-to-comparison, multi-voxel pattern analysis, cross-modal conjunction analysis

## Abstract

Neuroscientific research has shown that perceptual decision-making occurs in brain regions that are associated with the required motor response. Recent functional magnetic resonance imaging (fMRI) studies that dissociated decisions from coinciding processes, such as the motor response, partly challenge this, indicating that perceptual decisions are represented in an abstract or sensory-specific manner that might vary across sensory modalities. However, comparisons across sensory modalities have been difficult since most task designs differ not only in modality but also in effectors, motor response, and level of abstraction. Here, we describe an fMRI experiment where participants compared frequencies of two sequentially presented visual flicker stimuli in a delayed match-to-comparison task, which controlled for motor responses and stimulus sequence. A whole-brain searchlight support vector machine analysis of multi voxel patterns was used to identify brain regions containing information on perceptual decisions. Furthermore, a conjunction analysis with data from an analogue vibrotactile study was conducted for a comparison between visual and tactile decision-making processes. Both analyses revealed above-chance decoding accuracies in the left dorsal premotor cortex (PMd) as well as in the left intraparietal sulcus (IPS). While previous primate and human imaging research have implicated these regions in transforming sensory information into action, our findings indicate that the IPS processes abstract decision signals while the PMd represents an effector-dependent, but motor response independent encoding of perceptual decisions that is similar across sensory domains.

## 1 Introduction

Humans rely on a variety of sensory information to navigate the multitude of every day’s decisions. Thus, understanding the neural mechanisms of perceptual decision-making has been a fundamental inquiry of neuroscientific research that has been explored across various sensory modalities using diverse research paradigms. An important avenue of research has investigated the neural correlates of vibrotactile perceptual decisions in primates (LaMotte & Mountcastle, 1975;Romo & de Lafuente, 2013). These studies typically employ variants of delayed match-to-comparison (DMTC) tasks, where participants compare frequencies of two subsequently presented vibrotactile flutter stimuli and decide whether the frequency of the second stimulus (f2) was higher or lower than the frequency of the first stimulus (f1). The response is usually indicated with a button press. In their seminal work, Romo and colleagues have described perceptual decision-making in DMTC tasks as a sequence of processing steps that dynamically link action and perception. Following the initial encoding of f1 in somatosensory areas, information on stimulus frequencies is retained across different neurons in higher somatosensory and frontal areas (see below), showing opposite tuning curves for high and low frequencies. This mnemonic representation of f1 is maintained during the delay-period until the presentation of f2. Subsequently, as f2 is encoded in the same areas, the decision whether f2 was higher or lower than f1 likely arises through the generation of a difference signal between neurons whose firing can be described by opposite tuning curves (Romo & de Lafuente, 2013). Following this computation, which corresponds to a subtraction of the two frequencies, a binary signed decision signal emerges, which is then transformed into a motor command, observed within the primary motor cortex. Notably, alongside prefrontal (Jun et al., 2010) and secondary somatosensory regions (Romo et al., 2002), decision-related signals have been most prominently observed across different brain areas associated to planning and executing the required motor response. In the context of the vibrotactile DMTC task, these brain regions typically encompass motor-related areas involved in the planning and execution of button presses or arm movements. Accordingly, studies have reported decision-related activity in different sites of the premotor cortex, including the medial premotor cortex (PMm;de Lafuente & Romo, 2005;Hernández et al., 2002), the dorsal premotor cortex (PMd;Haegens et al., 2011;Rossi-Pool et al., 2017), and the ventral premotor cortex (PMv;Romo et al., 2004).

The findings by Romo and colleagues are in line with the intentional framework, which suggests that perceptual decisions are processed in brain areas associated with the required motor response (Shadlen et al., 2008). Similar to Romo and colleagues’ primate studies, human electroencephalography (EEG) studies using similar vibrotactile DMTC tasks revealed that beta-band amplitude in the premotor cortex (Herding et al., 2016) and parietal event-related potentials (Herding et al., 2019) were predictive of categorical choices. However, in most decision-making tasks, decisions are inextricably linked to task components such as the response, making it difficult to pinpoint neural markers of perceptual decisions independent of other sensory or motor processes. Consequently, it remains unclear whether these brain regions encode decisions as representations dependent on the required motor response or as a more general, effector-dependent representation that is independent of the motor response. While a motor response refers to the action or movement that is executed as the outcome of a decision (e.g., left vs. right button press or left vs. right saccade), an effector refers to the body part that is used to execute a response (e.g., the right hand for a button press or the eyes for a saccade). Thus, a motor response is more specific than an effector, but in most cases the two are inseparably linked. Therefore, to better understand the nature of these neural representations, it is necessary to decouple decision- from motor-related signals in the task designs. Human imaging studies that employed multiple effectors or flexible response-mappings to achieve such a decoupling have reported decision-related signals in varying brain regions, including abstract (e.g.,Filimon et al., 2013;Heekeren et al., 2006), sensory-modality specific (e.g.,Hebart et al., 2012;Liu & Pleskac, 2011), or effector-dependent representations (e.g.,Hebart et al., 2016;Y. Wu et al., 2019). These findings suggest that, if a decision is mapped to an abstract decision-rule, it is represented in higher-order brain regions, while a decision that is directly mapped to a specific response is encoded in brain regions associated with the required response. This notion was supported by an EEG study, which showed that beta-band power encoded decisions in the premotor cortex when the motor response was known, and in the parietal cortex when the decision was decoupled from the motor response (Ludwig et al., 2018). Interestingly, two recent vibrotactile DMTC studies that decoupled decisions from the response and the stimulus order in a functional magnetic resonance imaging (fMRI) experiment decoded categorical choices from brain regions that have been associated with planning and executing a required motor response, that is, frontal eye fields (FEF) for saccadic responses, PMd for button presses, and the intraparietal sulcus (IPS) for both (Y. Wu et al., 2019,^2021^). This indicates that these regions do not process perceptual decisions as concrete motor plans but instead in a more abstract, but effector-dependent manner, if a response effector is pre-specified.

To characterize the basic neural mechanisms underlying perceptual decision-making, it is crucial to examine the similarities and differences across sensory modalities. The sensory modality refers to the sensory modality of the stimulus or stimuli being evaluated to form a decision (e.g., vibrotactile for the flutter stimuli in DMTC tasks and visual for the motion stimuli in random dot motion tasks). Studies in different sensory modalities have causally linked perceptual decision processes to brain regions specific to the sensory modality of the stimulus, such as visual (e.g.,Hebart et al., 2012), auditory (e.g.Tsunada et al., 2016), and somatosensory cortices (Romo et al., 2002). These findings imply that perceptual decisions might be processed according to the low-level sensory features inherent to task stimuli. These low-level sensory processes are described as sensory evidence accumulation or comparison signals that are then conveyed to higher-order cortical regions relevant for decision formation and lastly for motor preparation and responses (Gold & Shadlen, 2007;Hernández et al., 2010;Tsunada et al., 2016). Importantly, comparing perceptual decisions across sensory modalities has been challenging due to distinct paradigms used to investigate perceptual decisions in different modalities, most notably differing in the applied motor responses. For instance, while vibrotactile decisions have been investigated using DMTC paradigms with button presses as the response modality, research on visual decisions has typically employed random dot motion tasks and saccades as the response effector (Gold & Shadlen, 2007;Shadlen et al., 1996). As described above, most of the perceptual decision literature suggests that perceptual decisions are represented in an intentional framework (Shadlen et al., 2008). Thereby, decisions evolve as motor intentions in brain regions associated with the required motor response and should be encoded independent of the sensory modality. Although earlier visual studies identified such decision-related signals from neurons in brain regions associated with planning and preparation of saccades, notably in the FEF and lateral intraparietal (LIP) neurons (Ding & Gold, 2012;Roitman & Shadlen, 2002;Shadlen & Newsome, 2001), newer findings suggest that these neurons represent aspects of decisions that occur independent of the motor response (Shushruth et al., 2022;So & Shadlen, 2022;Zhou et al., 2023). Furthermore, a human neuroimaging study that decoupled decisions from motor responses in a visual random dot motion task reported decision-related signals in the IPS (the human counterpart to LIP) and in the FEF (Liu & Pleskac, 2011). This indicates that IPS and FEF encode decisions according to sensory stimulus features (i.e., visual motion direction) and independent of the motor response (i.e., eye movement). In contrast, the vibrotactile studies byY. Wu et al. (2019,2021), who decoupled decisions from motor responses, reported decision-related activation patterns in brain regions associated with specific effectors, that is, FEF for saccades and PMd for button presses as well as in the IPS, suggesting an effector-dependent rather than a sensory-modality dependent representation. Importantly, these studies differ fromLiu & Pleskac (2011)as participants knew the effector modality they would use to select a response at the time of the decision. Thus, it is unclear whether these differences reflect inherent differences between the vibrotactile and visual modality or variations in task paradigms. Evidence from primate, single-unit recordings supports the latter, revealing functional heterogeneity in the FEF. While some neuron populations encode saccade preparation, others are tuned to sensory stimulus features, such as motion strength (Ding & Gold, 2012;Purcell et al., 2012). This suggests that both effector-dependent and effector-independent representations may coexist within the same brain regions. Despite these insights, no study has directly compared perceptual decisions across sensory modalities using tasks that explicitly control for stimulus features and separate decisions from motor-responses. To address this, it is crucial to use unified task designs that isolate perceptual decisions from other processes to identify underlying similarities and differences in neural processing underlying perceptual decisions across different sensory modalities.

The aim of the present fMRI study is to investigate cross-modal, that is, visual and vibrotactile, neural correlates of perceptual decisions, which are independent of motor responses and stimulus sequence. Therefore, we used a visual adaptation of the vibrotactile DMTC task ofY. Wu et al. (2021). To identify brain regions which process perceptual decisions, we applied a multi-voxel pattern analysis (MVPA) whole-brain searchlight approach (Kriegeskorte et al., 2006) using a support vector machine classifier (SVM). Furthermore, we conducted a conjunction analysis of both datasets to reveal how neural activation patterns reflect perceptual decisions across sensory modalities. We hypothesized effector-dependent activation patterns of perceptual decisions in both the left PMd and the left IPS. Moreover, we expected these regions to predict binary decisions consistently across the visual and the vibrotactile domain.

## 2 Methods

### 2.1 Participants

A total of 36 healthy volunteers participated in the fMRI experiment. Eligible participants were required to be right-handed, as assessed by the Edinburgh Handedness Inventory (Oldfield, 1971; 0.83 ± 0.18) and free from any neurological or psychiatric disorders. Data from 9 participants were removed due to strict exclusion criteria of behavioral performance (less than 50% correct responses in at least one stimulus pair). Another three participants were excluded due to a large amount of head movement (>3 mm), leaving 24 participants (13 males, 11 females) with a mean age of 24.8 (standard deviation [SD] = 3.5, range: 18–32) for further analysis. All participants provided written informed consent and were compensated monetarily for their participation. The study was approved by the local ethics committee of the Freie Universität Berlin (003/2021).

### 2.2 Task design and stimuli

**Fig. 1.**
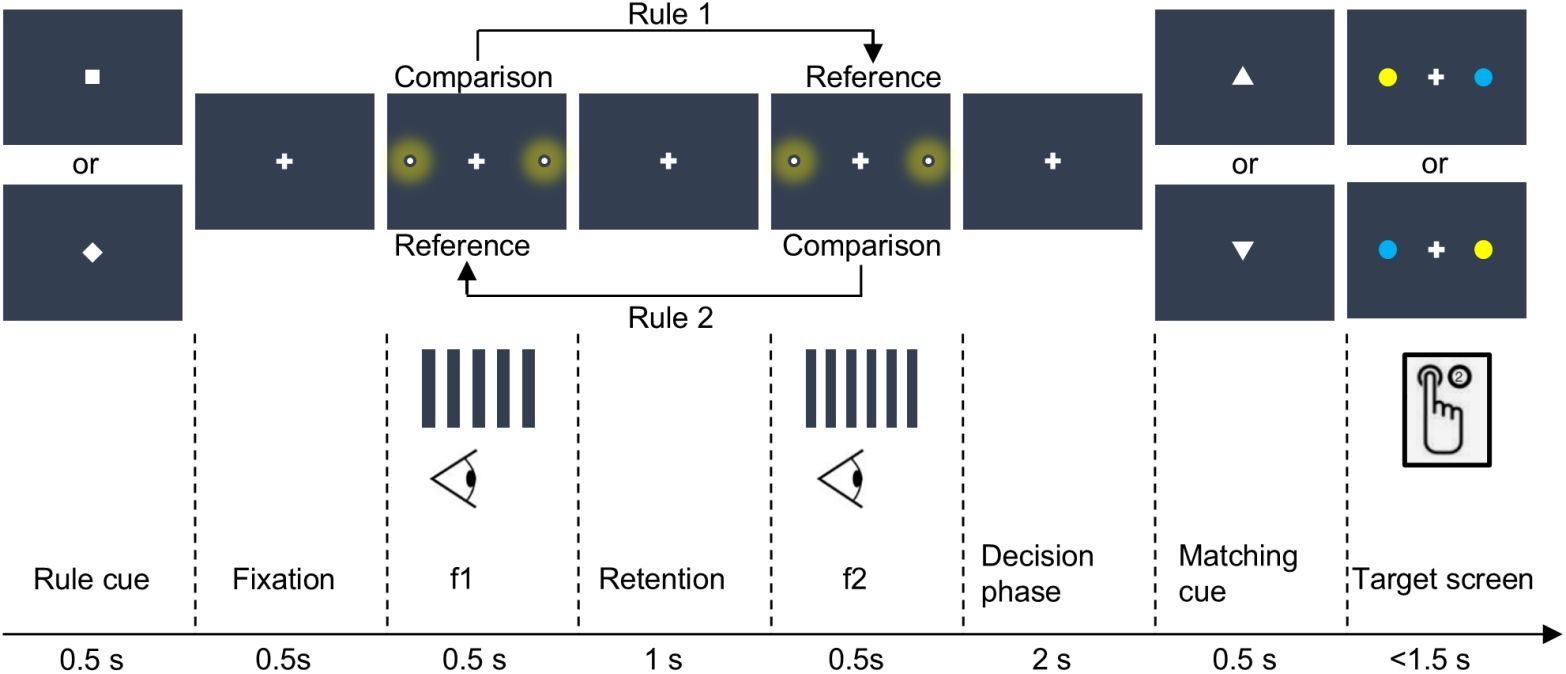
Experimental paradigm. An initial rule cue (square or diamond) indicated whether f1 would serve as the comparison and f2 as the reference stimulus (rule 1), or vice versa (rule 2). Subsequently, participants had to decide whether the frequency of the comparison stimulus was higher or lower than the frequency of the reference stimulus. Following a short fixation period, two visual flicker stimuli with differing frequencies were sequentially presented. After the decision phase, one of two matching cues was shown. An upward pointing triangle denoted that the frequency of the comparison stimulus was higher than the frequency of the reference stimulus, while a downward pointing triangle represented a lower frequency of the comparison stimulus. Participants then had to decide whether the matching cue correctly reflected their perception and indicate their decision with a button press associated to one of two colored disks on the following target screen.

The task design was adapted from the vibrotactile DMTC task byY. Wu et al. (2021). In our visual version of the DMTC task, we instructed participants to compare the frequency of two sequentially presented visual flicker stimuli (Fig. 1). f1 was 16, 20, 24, or 28 Hz and f2 was either 4 Hz above or below the first stimulus frequency (i.e., eight different frequency combinations in the frequency range of 12–32 Hz).

Trials started with the presentation of one of two rule cues (i.e.,Y. Wu et al., 2019,^2021^). Depending on its shape (square or diamond), the rule cue determined whether participants had to compare f1 against f2 or to compare f2 to f1 in half of the trials, respectively. The rule cue was introduced to decouple the decision (higher vs. lower) from potential effects of stimulus order (f1 > f2 vs. f1 < f2). After 0.5 s fixation period, two consecutive visual flicker stimuli were presented on both sides of the participants’ periphery (5°, eccentricity) for 0.5 s, separated by a retention interval of 1 s. Following a 2-s decision phase, a matching cue was presented, consisting of a triangle pointing either upward or downward. Participants had to compare their decision to the orientation of the matching cue (an upward pointing triangle meaning “higher”, downward meaning “lower”), to decide for “match” (the matching cue was consistent with their stimulus comparison, i.e. true) or “mismatch” (the matching cue was inconsistent with their stimulus comparison, i.e. false). This procedure was introduced to render decisions independent from the motor response to avoid response-related confounds. The matching cue was independent from the true frequency difference, and matches/mismatches were balanced within each run. Finally, participants reported whether the comparison between their perception and the matching cue resulted in a match or mismatch (i.e., the matching cue was true or false) during the presentation of a target screen, displayed until the response but for a maximum of 1.5 s. The target screen comprised a central fixation cross and two coloured target disks (blue and yellow) in the periphery along the horizontal meridian (3°, eccentricity). The colour assignment for match or mismatch was balanced across participants. Depending on the location of the target, participants responded with a left or right button press, using their right-hand index or middle finger, rendering the motor response independent of the perceptual decision. The target side was balanced within a run. Inter-trial intervals with a fixation period of varying durations (3, 4, 5, or 6 s) were administered between all trials.

During the fMRI session, the visual cues were projected onto a screen on the bore opening of the MR scanner. Participants viewed the visual displays through a mirror attached to the MR head coil from approximately 110 ± 2 cm. The cues were presented with MATLAB version 9.13 (The MathWorks, Inc, Natick, MA) using Psychtoolbox-3 (Brainard, 1997). Visual flicker stimuli were generated using a sine function with a fixed voltage amplitude of 10 V. In each trial, the sine waves were received and stored by a data acquisition card (NI-USB 6343; National Instruments Corporation, Austin, Texas, USA) and released upon a trigger signal to ensure precise timing. The visual flicker stimuli were presented with light emitting diodes (LEDs), transmitted through fiber-optic cables, and presented 10 cm to the left and right of a fixation cross on both sides of the screen. The LEDs illuminated above a threshold of 2.24 V such that the duty cycle of the flicker stimuli was approximately 43%.

After training the task for 20–40 min, participants performed six experimental runs inside of the fMRI scanner on a separate day. A run lasted approximately 12.5 min and consisted of 64 trials with each of the eight frequency combinations being presented eight times per run. Each of the eight presentations contained a unique combination of rule cue, matching cue as well as target screen.

### 2.3 fMRI data acquisition and preprocessing

Functional magnetic resonance imaging (fMRI) data were acquired on a 3 T Magnetom Prisma Fit Scanner (Siemens Healthcare GmbH, Erlangen, Germany) at the Center for Cognitive Neuroscience Berlin, using a 32-channel head coil. In each of the six experimental runs, 378 functional, T2*-weighted volumes were acquired with a repetition time (TR) of 2000 ms, an echo time (TE) of 30 ms, an in-plane resolution of 64 x 64, a flip angle of 70°, and a voxel size of 3 x 3 x 3 mm³. Furthermore, a T1-weighted image with 176 sagittal slices was acquired (TR = 1900 ms, TE = 2.52 ms, in-plane resolution: 256 x 256, voxel size: 1 x 1 x 1 mm³).

### 2.4 fMRI analysis

Pre-processing and general linear model (GLM) analysis of the fMRI data was performed with SPM12 version v7388 (http://fil.ion.ucl.ac.uk/spm/). During the pre-processing, the functional images were slice-time corrected, realigned to the mean image, and co-registered with the structural image.

To estimate voxel-wise decision-related activity patterns, we fitted a GLM with a 192 s high-pass filter to each participant’s functional data. Within each GLM, we estimated run-wise beta estimates during the decision phase for all voxels. The two regressors of interest included the categorical outcome of correct decisions (“higher” vs. “lower”) and were convolved with the hemodynamic response function at the onset of the decision phase. Incorrect decisions were modeled with a separate regressor of non-interest. Additionally, six movement parameters, the first five principal components explaining variance in the white matter and cerebrospinal fluid signals respectively (Behzadi et al., 2007) and a run constant, were added as nuisance regressors. Altogether, this yields 20 regressors for each run, resulting in a total of 120 regressors.

To identify brain regions with activation patterns being predictive of categorical decisions, we applied an MVPA whole-brain searchlight approach for each participant. Voxel-wise activation patterns with above-chance decoding accuracies were obtained with an SVM classifier using version 3.999F of The Decoding Toolbox (TDT;Hebart et al., 2015). A searchlight radius of 4 voxels was used to match the prior study byY. Wu et al. (2021). However, the consistency of the main findings was additionally tested through control analyses with searchlight sizes of 3- and 5-voxel radius. Run-wise beta estimates of the GLM analysis were retrieved for all incorporated voxels of each searchlight. Then, a six-fold leave-one-run-out cross-validation procedure was applied, as implemented in TDT (Hebart et al., 2015). The resulting prediction accuracy maps comprised above-chance decoding accuracies at each searchlight location, depicting the ability to accurately predict the decision (higher vs. lower). For the subsequent group-level analyses, the single-subject correlation maps were normalized to MNI space, resampled to a voxel size of 2 × 2 × 2 mm³, and spatially smoothed using a 3 mm full width at half maximum Gaussian kernel. Correlation maps were then entered into a one-sample t-test, to test for local brain activation patterns that showed significant above-chance decoding accuracies on the group level. The results are presented at a voxel-level threshold of*p*< 0.001, corrected at the cluster-level using family-wise error (FWE) correction at*p*< 0.05. The anatomical regions were identified with the JuBrain Anatomy Toolbox (Eickhoff et al., 2005).

### 2.5 fMRI control analyses

We conducted two additional decoding analysis to identify brain regions encoding information about the motor response and task rule during the decision. To this end, we implemented two separate GLMs with regressors modeling the motor response and task rule at the onset of the decision phase (seeFig. 1). Similar to our main analysis, nuisance regressors were included to control for movement-related effects and variance in white matter and cerebrospinal fluid signals. Subsequently, two MVPA whole-brain searchlight approaches with a searchlight radius of 4 voxels were applied to the resulting beta images. An SVM classifier using a six-fold leave-one-run-out cross-validation procedure was employed to find brain activity patterns with above-chance decoding accuracies for motor responses and task rules.

To determine whether the reported brain regions encode categorical decisions independently of action planning and selection (left vs. right) and the representation of specific task rules (first against second vs. second against first), we conducted an additional neuroimaging control analysis. Specifically, we repeated the main decoding analysis, while balancing both response direction and task rule across conditions and runs. This was accomplished through a subsampling scheme in which the searchlight decoding analysis was repeated 100 times. For each subsample, a subset of trials was randomly selected to ensure that both response direction and task rules would occur equally as often across choices and runs. The resulting 100 accuracy maps were averaged into a single global accuracy map per participant, which was subsequently used for the second-level analysis. If the results of these subsampling analyses aligned with our main results, this would indicate that our findings were not confounded by motor- or rule-dependent activation.

### 2.6 Cross-modal analysis

One of our main objectives was to investigate the neural underpinnings of perceptual decision across sensory modalities. Therefore, we implemented a conjunction analysis with the vibrotactile data obtained byY. Wu et al. (2021), who used an identical study design as well as identical fMRI analysis parameters. To identify cross-modal decision-specific activation patterns, we entered the first-level accuracy maps from both studies into a flexible factorial second-level model. Then, we computed a conjunction analysis to test the results against the conjunction null hypothesis (Nichols et al., 2005) and reported the results at an uncorrected threshold (*p*< 0.001). We would, therefore, like to point out that these results, which are not corrected for multiple comparison, must be viewed with caution. However, the high spatial specificity of the results found exclusively in a priori assumed brain areas and the fact that the conjunction null hypothesis is known to be overly conservative (Friston et al., 2005) supports the decision to report these results. In addition to this conservative conjunction approach, a more liberal approach was also implemented by testing against the global null hypothesis (Price & Friston, 1997). However, in this analysis, FWE-correction was applied.

## 3 Results

### 3.1 Behavioral results

Participants performed the task with a mean accuracy of 87.6 % (SD: 5.4 %, range: 77.3–96.1 %) and an average response time of 506 ms (SD: 74 ms, range: 389–632 ms). To compare behavioral performances toY. Wu et al. (2021), we used a two-sample z-test of proportions as well as a two-sample t-test to compare response times. The tests revealed no significant difference of mean accuracies (Δ_accuracies_= 0.4 %,*p*= 0.964), while response times were slightly lower in our task (Δ_RTs_= -60 ms,*p*= 0.024). Overall, the task difficulty was approximately equal between both studies. To assess the effects of rule (compare f1 against f2 vs. f2 against f1), stimulus order (f1 > f2 vs. f1 < f2), and f1 frequency (16 Hz, 20 Hz, 24 Hz, and 28 Hz) on the performance, we implemented a three-way repeated measure analysis of variance (ANOVA). To account for the bounded nature of proportional data, we applied an arcsine transformation to stabilize variance and meet the assumptions of normality required for an ANOVA. The ANOVA revealed substantial performance differences across conditions (seeFig. 2). Similarly toY. Wu et al. (2021), there was no significant main effect of task rule (F(1,23) = 3.626,*p*= 0.07) and a significant effect of stimulus order (F(1,23) = 60.755,*p*< 0.001). However, in contrast toY. Wu et al. (2021), this main effect was much stronger, and its direction was reversed, with a better performance in f1 < f2 trials (mean = 92.8 %) than in f1 > f2 trials (mean = 82.3 %). Furthermore, there was a significant interaction between task rule and stimulus order (F(3,69) = 5.063,*p*= 0.034), indicating that the difference between f1 < f2 trials and f1 > f2 trials would be slightly higher for rule 2 than for rule 1. Similarly toY. Wu et al. (2021), the performance decreased with increasing f1 only in f1 > f2 trials while the performance in f1 < f2 trials remained the same throughout f1 frequencies, indicated by a significant main effect of f1 frequencies (F(1,23) = 15.972,*p*< 0.001) as well as a significant interaction between stimulus order and f1 frequencies (F(3,69) = 14.389,*p*< 0.001).

**Fig. 2.**
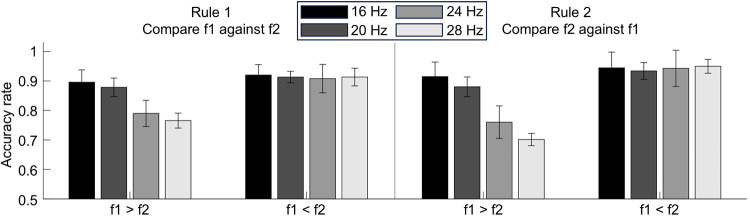
Behavioral results. The bar plots show the average performance across participants over all runs for different stimulus orders, rules, and f1 frequencies. Error bars show the 95% confidence intervals (CIs).

We further tested whether potential biases in left and right motor responses within participants could have distorted the main decoding results. Note that the required motor responses were balanced through the task design. To that end, we computed Pearson chi-square tests comparing the distribution of the two conditions “higher” vs. “lower” (indicating whether the true comparison frequency was higher or lower than the reference frequency) between left and right motor responses, for each participant. The results showed a significant difference for one participant (*p*= 0.045). In the remaining 23 participants, no significant difference was observed (all*p*> 0.1), suggesting that our main results were not influenced by imbalanced motor responses.

### 3.2 fMRI results

In the current study, our primary aim was to identify brain regions that convey information about perceptual decisions, irrespective of stimulus order and motor responses. To accomplish this, we applied an MVPA, whole-brain searchlight approach during the 2-s decision phase. This approach allowed us to systematically test for brain regions that are predictive of the binary decision (high vs. low). The results of the SVM, as depicted inFigure 3, revealed 6 clusters, mainly located in contralateral parts of premotor and parietal cortices, that showed local activation patterns predicting perceptual decisions irrespective of stimulus order and motor response (FWE corrected at the cluster level at*p*< 0.05). These included clusters in the left PMv (peak voxel: [-44 6 2]; cluster size: 1627), the left IPS (peak voxel: [-38, -40 58]; cluster size: 1619), the left superior parietal lobule (SPL; peak voxel: [-10 -64 40]; cluster size: 914), the right posterior anterior cingulate cortex (pACC; peak voxel: [24 54 16]; cluster size: 301), the left PMd (peak voxel: [-8 -14 76]; cluster size: 221), and the right SPL (peak voxel: [14 -46 72]; cluster size: 205). For comprehensive details refer toSupplementary Table 1. To further solidify the robustness of our findings, we repeated the SVM with different searchlight radii of 3 and 5 voxels, respectively. Both different searchlight radii reaffirmed the involvement of the same brain regions.

**Fig. 3.**
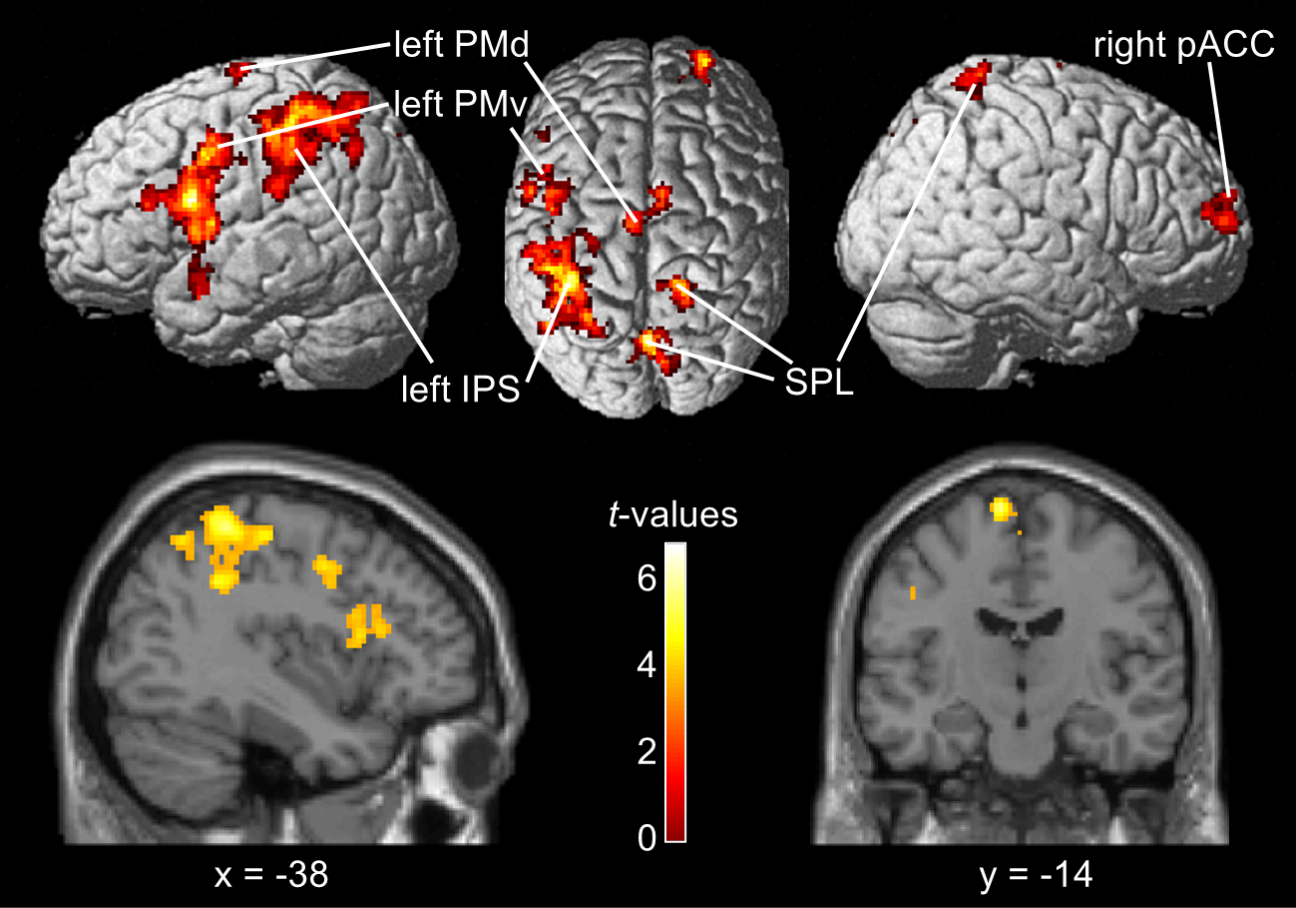
Results of the SVM analysis. The SVM revealed activation pattern in the left IPS, left PMd, left PMv, right pACC, as well as bilateral SPL that were predictive of categorical decisions. Results are displayed at a voxel-level threshold of*p*< 0.001, FWE corrected on the cluster-level at*p*< 0.05. The unthresholded statistical map is accessible viahttps://neurovault.org/collections/WILTPYNG/images/899935/.

To test for differences in prediction accuracy between hemispheres for IPS, SPL, PMv, and PMd, single subject prediction accuracies were extracted from the peak voxel in the respective region of each hemisphere and compared with one-sided paired t-tests, hypothesizing higher prediction accuracies in the left hemisphere, contralateral to the effector. The t-test comparing left versus right IPS did not reveal a significantly higher decoding accuracy in the left IPS (t(23) = 1.131,*p*= 0.269). The same was true for left versus right SPL (t(23) = 0.655,*p*= 0.519) and left versus right PMd (t(23) = -0.252,*p*= 0.598). After Bonferroni correction, there was also no significantly higher prediction accuracy in the left versus right PMv (t(23) = 1.727,*p*= 0.034).

### 3.3 fMRI control analyses

We additionally employed two SVM searchlight analyses to test for brain activation patterns with above-chance decoding accuracies of motor response and task rule during the decision period. Activation patterns selective for the motor response (left vs. right) were observed in the left primary motor cortex as well as in the bilateral occipital cortex. Activation patterns selective for the rule (compare f1 against f2 vs. f2 against f1) were observed in the bilateral PPC and in the bilateral premotor cortex.

We conducted an additional control analysis to eliminate the possibility that our results were biased by imbalances in the distributions of motor responses or task rules. To achieve this, we repeated the main searchlight decoding analysis while subsampling trials to ensure that response direction and task rules were balanced across decisions and runs. Despite the significant data reduction, this analysis yielded highly similar results to those of our main analysis with significant clusters in the left PMv, left PMd, and bilateral PPC (seeSupplementary Fig. 1).

### 3.4 Cross-modal analysis

The second aim of our study was to identify brain regions that carry information on perceptual decisions across different sensory domains. To achieve this, we conducted a conjunction analysis against the conjunction null hypothesis (Nichols et al., 2005) to compare our results to data of a vibrotactile study (Y. Wu et al., 2021), which used an identical task design. Specifically, we tested the first-level accuracy maps of both studies in a conjunction analysis to assess which brain regions predicted categorical perceptual decisions throughout both modalities. The conjunction analysis revealed above-chance decoding accuracies in the left IPS (peak voxel: [-34 -54 56], cluster size = 85), the left primary motor cortex (peak voxel: [-36 -36 54], cluster size = 54), and the left PMd (peak voxel: [-10 -14 72], cluster size = 6). Please note that these results are reported at a significance threshold of*p*< 0.001, without correction for multiple comparisons. The results of the conjunction analysis are depicted inFigure 4. For comprehensive details of the regions reported from the conjunction refer toSupplementary Table 2. To further validate our findings, we also performed a conjunction analysis as a test against the global null hypothesis (Price & Friston, 1997). This more sensitive approach confirmed the results from the conjunction null analysis, revealing above-chance decoding accuracies in the bilateral IPS and left PMd, among other regions (seeSupplementary Fig. 2andSupplementary Table 3). However, although these results indicate that the effects were consistently high and jointly significant, they do not strictly imply that both contrasts were individually significant (Friston et al., 2005). Overall, the brain regions involved in encoding perceptual decisions showed substantial overlap across sensory domains, indicating that decisions are encoded as effector-dependent neural representations, that are at least partially independent of the specific sensory modality.

**Fig. 4.**
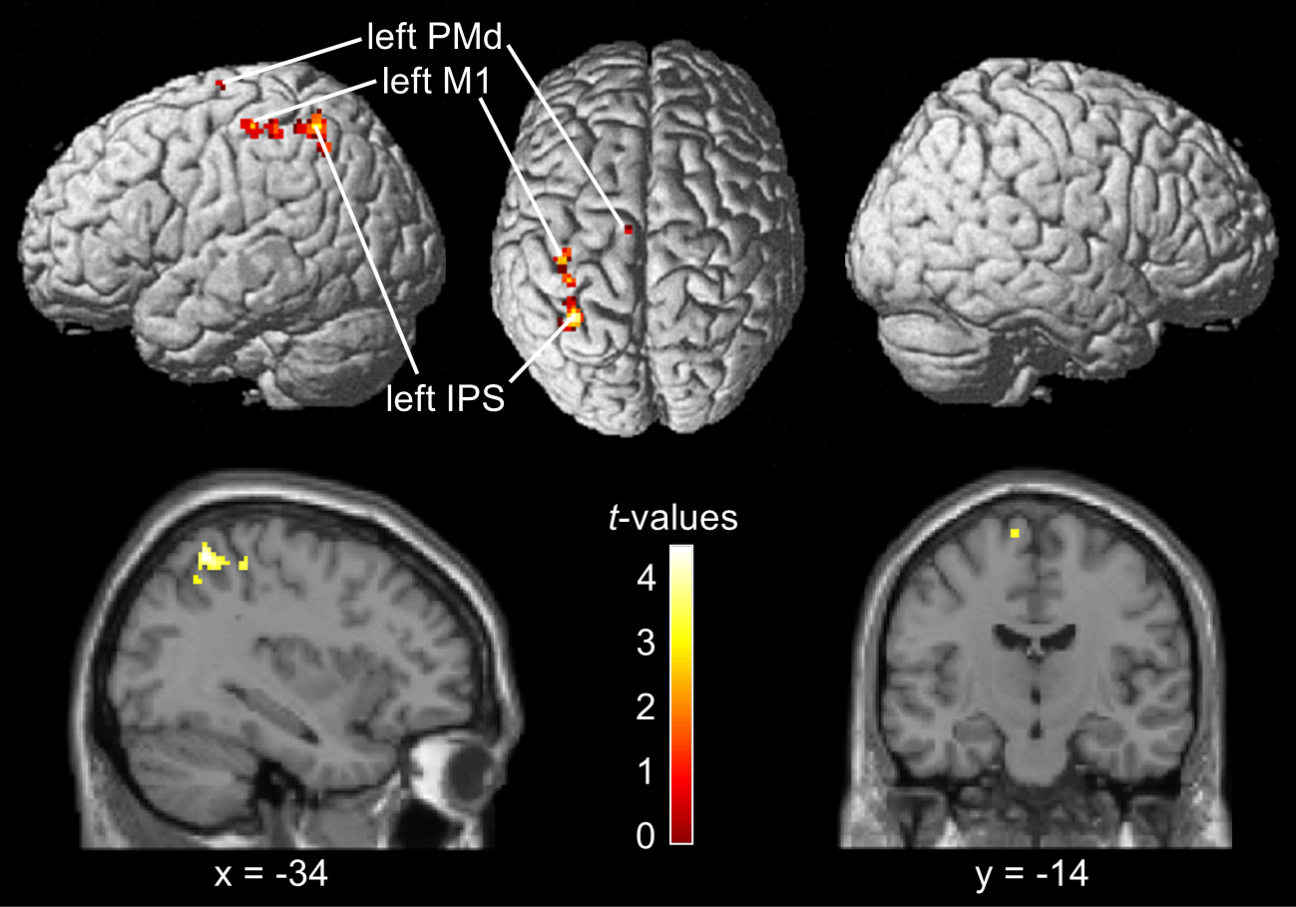
Results of the conjunction analysis between our visual data and the vibrotactile data obtained byY. Wu et al. (2021), tested against the conjunction null hypothesis (Nichols et al., 2005). The conjunction revealed above-chance decoding accuracies in the left IPS, the left primary motor cortex (M1), and the left PMd (subregion 6d1 according to the JuBrain Anatomy toolbox,Eickhoff et al., 2005). Results are displayed at a voxel-level threshold of*p*< 0.001 (uncorrected). The unthresholded statistical map is accessible viahttps://neurovault.org/collections/WILTPYNG/images/896900/.

## 4 Discussion

In this fMRI study, we aimed to test for effector-dependent neural correlates of decisions in the visual domain that are independent from the required motor response. Thus, we used a visual version of a DMTC task that rendered decisions independent from choice direction and response selection. Thereby, the right hand was pre-specified as the effector, while the motor response—a left or right button press—was unknown during the decision process. The SVM MVPA searchlight approach revealed above-chance decoding accuracies in the left PMd, the left PMv, the left IPS, the right SPL, and the right pACC. Using the same task design and analysis parameters, a similar vibrotactile DMTC study fromY. Wu et al. (2021)reported above-chance decoding accuracies in the left PMd and the left IPS, suggesting a substantial cross-modal overlap between visual and vibrotactile sensory domain. To identify the cross-modal neural correlates of perceptual decision, we computed a conjunction analysis against the conjunction null hypothesis, which was not corrected for whole-brain multiple comparisons, and a conjunction against the global null hypothesis with their data and the present data. Both revealed above-chance decoding accuracies in the left PMd and the left IPS. Altogether, activation patterns in the left PMd and the PPC, particularly in the IPS, showed consistent overlap in predicting perceptual decisions across sensory modalities, irrespective of stimulus order and motor response. While these findings align with the hypothesized regions, suggesting that effector-dependent representations of perceptual decisions may generalize across sensory modalities, the lack of whole-brain correction in the conjunction against the conjunction analysis as well as the limited interpretability of the conjunction against the global null hypothesis should be taken with caution. Nonetheless, alongside previous findings byY. Wu et al. (2019,2021), our results support the hypothesis of an effector-dependent encoding of perceptual decisions that is not only independent of task-related processes but that also generalizes across sensory modalities.

### 4.1 The role of the premotor cortex in perceptual decision-making

The premotor cortex has been found to play a crucial role in perceptual decisions by a series of primate vibrotactile studies which used arm movements or button presses as responses (reviewed inRomo & de Lafuente, 2013). The activity within premotor regions has been linked to different stages of the decision-making process, likely reflecting the integration of mnemonic processes and sensory inputs towards the formation of behavioural responses (e.g.,Hernández et al., 2002;Wallis & Miller, 2003). Results from primate studies suggest that the PMd and PMv is involved in transforming sensory information into action through an evidence accumulation process (e.g.,Cisek & Kalaska, 2005;Lemus et al., 2009;Romo et al., 2004;Wang et al., 2019). While the PMv has been associated to processes linked to action selection, the PMd has been more strongly associated with action preparation (e.g.,Pardo-Vazquez et al., 2008;Pardo-Vázquez et al., 2011). Similarly toY. Wu et al. (2021), our study decoupled the decision from task-related processes, notably motor responses and stimulus order, to identify the precise role of premotor cortices in perceptual decision-making. Both our study and the findings fromY. Wu et al. (2021)indicate that the left PMd is involved in decision-making, irrespective of these task-related processes, supporting the notion that the PMd encodes categorical decisions. In addition to the original findings byY. Wu et al. (2021), we found above-chance decoding accuracies in the left PMv. However, the PMv was not predictive of perceptual decisions in either of the conjunction analyses. Furthermore, in our study, the peak of the PMd was slightly more posterior and medial compared toY. Wu et al. (2021). This, alongside the lack of whole-brain correction for multiple comparisons in the conservative conjunction analysis, suggests that while effector-dependent overlaps are evident, cross-modal differences may also exist in how decisions are represented within the premotor cortices. As hypothesized, the results of our main analysis and conjunction suggest a lateralized encoding of decision-related information in the premotor cortex contralateral to the effector. Although significant clusters were only observed in the contralateral hemisphere of PMv and PMd, a comparison of peak-voxels within PMd and PMv did not reveal a significantly higher decoding accuracy in the contralateral hemisphere. Thus, it remains possible that the more abstract effector-dependent representations investigated in our study could be represented partially bilaterally. This suggests that the observed activation patterns in premotor cortices might not be purely specific to an effector but rather represent more complex, associative processes that involve perception, working memory, and categorical decisions as suggested byRossi-Pool et al. (2017). Overall, our findings support the relevance of the premotor cortex in perceptual decision-making, even when participants have no knowledge about the upcoming motor response if the effector is pre-specified. Although our study suggests an effector-dependent but motor-response independent encoding of perceptual decisions, it needs further research to elaborate the precise role of the premotor cortex.

### 4.2 The role of the posterior parietal cortex in perceptual decision-making in primates

A substantial body of primate studies investigating the neural correlates of perceptual decision-making has focused on the role of the PPC, with particular emphasis on LIP neurons (Gold & Shadlen, 2007). Most visual decision studies suggested that LIP neurons are predominantly involved in an effector-dependent evidence accumulation process among competing saccade responses (Roitman & Shadlen, 2002;Shadlen & Newsome, 2001). However, almost all these studies used visual random dot motion tasks with saccades as response effectors, making it difficult to draw clear conclusions on the precise involvement of posterior parietal regions. Accordingly, recent primate studies have challenged this notion, showing that LIP neurons encoded perceptual decisions even in situations where the response was unpredictable during stimulus presentation (Shushruth et al., 2022) or disrupted (So & Shadlen, 2022). Freedman and colleagues conducted a series of studies employing a delayed match-to-category task with arbitrary categories of different random dot motion stimuli and manual arm movements as response modalities. Their results indicated that activity in LIP neurons reflect categorical decisions independent of specific effectors or sensory modalities (Freedman & Assad, 2006;Swaminathan & Freedman, 2012;Swaminathan et al., 2013). Furthermore, by pharmacological inactivation, they demonstrated that LIP neurons have a causal role in evaluating task-relevant sensory stimuli that goes beyond the previously suggested primary function of merely representing motor responses (Zhou & Freedman, 2019). Interestingly, LIP inactivation impaired performance levels across different decision-making tasks regardless of the response modality used (Zhou et al., 2023). Overall, findings from primate studies suggest that neuronal activity in the PPC reflects decisions beyond a specific effector. Thus, despite its repeatedly demonstrated involvement in perceptual decision-making, the precise role of the PPC remains unclear.

### 4.3 Sensorimotor mapping in the posterior parietal cortex

Similar to the abovementioned primate studies, human neuroimaging studies also challenge an exclusively effector-specific role of the PPC in perceptual decision-making. For instance, different regions of the PPC exhibited sustained activity throughout both arm reaching and saccadic responses, albeit with local variations in effector preference (Levy et al., 2007). Along these lines, a recent behavioral study on visual learning showed that training effects were only partially transferred between saccadic and reach responses. This partial learning transfer suggests that perceptual learning is neither entirely effector-dependent nor completely effector-independent but rather entails a sensorimotor mapping from visual regions to effector-dependent integrator regions. Given the PPC’s localization within the sensorimotor hierarchy as well as its overlapping encoding of multiple effectors, it emerges as a likely candidate region for a sensorimotor mapping across effectors (Ivanov et al., 2024). This notion was further supported by findings from the vibrotactile DMTC studies byY. Wu et al. (2019,2021), who showed that the IPS was predictive of binary decisions regardless of whether the effector was saccades or button-presses. Our results replicate these findings in a perceptual decision task in a different sensory modality, indicating that the IPS does not only represent decisions beyond the motor response but also independent of the stimulus modality. Overall, this implies that the PPC conveys an abstract decision variable across multiple domains, with a varying degree of effector-dependence.

### 4.4 Effector-dependent representations across sensory modalities

As described in the sections above, findings from primate studies overwhelmingly provide evidence for fully and partly effector-dependent representations of visual decisions in premotor and posterior parietal regions. Conversely, when decoupling the decision from the motor response, human imaging studies suggest that decisions tend to be represented either in a more abstract manner in posterior parietal and prefrontal brain regions (e.g.,Filimon et al., 2013;Heekeren et al., 2008) or in brain areas associated with sensory specific stimulus features (Liu & Pleskac, 2011). In contrast, when decoupling decisions from the motor response in a vibrotactile DMTC task,Y. Wu et al. (2019,2021)demonstrated that activation patterns in brain regions associated with specific effectors (in the FEF for saccadic responses and in the PMd for button press responses) were still predictive of categorical decisions. Comparing these findings to studies in the visual domain (e.g.,Liu & Pleskac, 2011) indicates that there might be an interaction between effector and stimulus modality. However, using a visual DMTC task with button presses as the response, our results revealed activation patterns in highly similar brain regions linked to specific effectors that also persisted in the cross-modal comparison between the visual and vibrotactile stimulus domains, although in slightly different subregions of the PMd and at an uncorrected threshold in the strict conjunction. While this overlap provides evidence for an effector-dependent component in the encoding of perceptual decisions, it is important to consider the heterogeneity of neural responses within these regions. For instance, the PMd, while associated with finger-based responses (e.g., button presses), has also been linked to other effectors, such as the arm for reaching (Hoshi & Tanji, 2002), the hand for grasping (Caccialupi et al., 2025) and the foot for foot movements (Sahyoun et al., 2004), albeit with different subregions involved. Moreover, responses in these brain regions have also been implicated in effector-independent processing of sensory or abstract information during perceptual decisions. For instance, primate studies have shown that while some single-units in the FEF are tuned to effector-dependent features, others encode sensory attributes like stimulus movement (Ding & Gold, 2012;Purcell et al., 2012). Since fMRI does not provide the resolution needed to distinguish between neuronal populations within subregions, our results cannot confirm or rule out a purely effector-specific representation. Nonetheless, the considerable similarity between the representations of vibrotactile and visual modality in these regions, combined with the orthogonalization of decisions from motor responses and sensory task attributes in the task design, suggests a substantial effector-dependent component. This interpretation is further supported by comparing our results to a similar vibrotactile study byY. Wu et al. (2019), which used eye movements as the required motor response and reported above-chance decoding accuracies in the FEF but not in the PMd. Additional insight can be provided from the mouse model byZ. Wu et al. (2020). They used optogenetics to inactivate premotor areas in an olfactory delayed match to sample task. Similar to our study, the mice knew the effector modality but were not able to plan a specific motor movement before the response. The disruption of neurons in the premotor cortex impaired task performance, suggesting that the relevance of premotor regions extend beyond the preparation of movements. Along with our results, it appears that the effector-dependent encoding of perceptual decisions in brain regions classically associated with planning and executing specific movements does not strictly constitute a mapping that is bound to a specific motor-movement. Rather, effector-dependent representations in these brain regions appear to retain abstract information about stimulus identity and subsequently transforming it into motor responses.

### 4.5 Flexible representations of perceptual decision processes

The findings from human studies seem to be somewhat incoherent, with some of them supporting an effector-dependent representation of perceptual decisions while others suggest more abstract or sensory-modality specific representations (Liu & Pleskac, 2011). However, upon further examination, this apparent discrepancy may be attributed to fundamental differences in analysis methods and task design. Firstly, our study employed a multivariate approach that aims towards uncovering nuanced local cortical activation patterns instead of focusing solely on average BOLD-signal. Consequently, distinct patterns that rely on both activation and deactivation may have been averaged out in studies that use conventional fMRI analyses. Secondly, and perhaps more importantly, our study rendered decisions independent of a specific motor response while still preserving dependence on the effector itself. In contrast, prior studies that aimed to disentangle motor responses and decisions relied on abstract associations between decision and motor response, wherein participants were unaware of the effector until making their response. Overall, this implies that decisions are represented flexibly, depending on task demands. When a task does not pre-specify an effector, a decision between stimuli cannot be directly mapped to brain regions involved in planning and executing the respective motor responses. Instead, information remains within brain areas associated with the sensory modality (Liu & Pleskac, 2011) or is transferred to a central, more abstract decision hub, for example, in posterior parietal regions (O’Connell et al., 2012;Sandhaeger et al., 2023). On the other hand, if an effector is pre-specified, although a decision cannot be directly transformed into a specific motor response, decisions are categorized as abstract motor intentions in brain regions associated with the required effectors, facilitating the transformation into specific motor plans and actions upon presentation of the response mapping.

### 4.6 Conclusion

In conclusion, our fMRI study suggests that if a response effector is pre-specified, local brain activation patterns encode perceptual decisions in an effector-dependent manner. Notably this encoding remains independent of stimulus order, motor response, and likely sensory modality. It is conceivable that the PMd processes such effector-dependent decision signals, while the IPS adopts a more abstract representation of perceptual decisions which persists across different effectors. Overall, our findings support the notion of a cross-modal, effector-dependent representation of perceptual decisions if an effector is pre-specified, although there may be variations in how these representations manifest across different subregions. Being the first study comparing perceptual decisions across different sensory modalities in an equivalent experimental task that controls for motor- and task-related confounds, our study contributes to a deeper understanding of perceptual decision-making across a variety of sensory contexts.

## Supplementary Material

Supplementary Material

## Data Availability

The data that support the findings of this study are available on request to M.F.E. (marlon.esmeyer@fu-berlin.de). In accordance with EU’s General Data Protection Regulation and specifications in data protection section of the participants consent forms, we cannot share raw fMRI data. However, group-level statistical maps are available at:https://neurovault.org/collections/WILTPYNG/. All analysis scripts are available at:https://github.com/marlones95/vDMTC.git*.*
