# Supplementary material for: Effector-dependent neural representations of perceptual decisions independent of motor actions and sensory modalities
Source: Imaging Neurosci (Camb). 2025 May 22;3:IMAG.a.11. doi: 10.1162/IMAG.a.11 (PMC12319951; doi:10.1162/IMAG.a.11)
Supplement: Supplementary Material [file imag.a.11_supp.pdf]

Supplementary Material

This supplementary material accompanies the article:

Title: "Effector-Dependent Neural Representations of Perceptual Decisions Independent of Motor Actions and Sensory Modalities"

Authors: Marlon F. Esmeyer, Timo T. Schmidt, Felix Blankenburg

Supplementary Table 1. Brain regions predictive of binary decisions in the visual DMTC task.

		**Peak MNI coordinates**				
**Anatomical regions**	**Cluster size**	**x**	**y**	**z**	***t*-value**	**Accuracy**
Left PMv (Area 44)	1627	-44	6	22	6.8	0.612
Left IPS (hIP2, hIP1), left SPL (7A), left IPL (PFt), left Area 2	1619	-38	-40	58	5.63	0.627
Right SPL (7M)	914	-10	-64	40	5.41	0.603
Right pACC (p24c)	301	24	54	16	5.14	0.605
Left PMd (6d1), right SMA (6mc)	221	-8	-14	76	5.69	0.586
Right SPL (5L)	205	14	-46	72	4.93	0.601

The table depicts all significant clusters of the SVM searchlight analysis. Results are reported at a threshold of $p < 0.001$, FWE corrected on the cluster-level at $p < 0.05$.

Supplementary Table 2. Brain regions predictive of binary decisions across sensory modalities testing against the conjunction null hypothesis.

		Peak MNI coordinates			
**Anatomical regions**	**Cluster size**	**x**	**y**	**z**	***t*-value**
Left IPS (hIP3), left SPL (7PC)	85	-34	-54	56	4.71
Left Area 4p, left Area 2, left Area 3b	54	-36	-36	54	3.9
Left PMd (6d1)	6	-10	-14	72	3.46
Right Hippocampus (CA1)	3	22	-44	4	3.5
Left Insula (Id6)	2	34	6	10	3.34
Left Precuneus	1	-10	-52	22	3.31

The table depicts all significant clusters of the conjunction analysis. Results are reported at a voxel-level threshold of $p < 0.001$ (uncorrected).

Supplementary Table 3. Brain regions predictive of binary decisions across sensory modalities testing against the global null hypothesis.

		Peak MNI coordinates			
**Anatomical regions**	**Cluster size**	**x**	**y**	**z**	***t*-value**
Left IPS (hIP3, hIP1, hIP6), left Area 4p, left Area 3b	2303	-34	-54	56	4.71
Left PMd (6d2, 6d1)	771	-10	-14	72	3.46
Right Hippocampus (CA1, DG)	381	22	44	4	3.5
Left PFC (Fp1)	281	-26	64	4	3.11
preSMA (6mr)	206	2	10	58	2.76
Right IPS (hIP4, hIP7)	168	38	-76	30	2.84
Right V3d (hOc3d)	141	6	-72	32	2.71
Left Insula (Id6)	121	-34	6	10	3.34
Right IPS (hIP1)	90	36	-36	34	2.88

The table depicts all significant clusters of the conjunction analysis. Results are reported at a threshold of $p < 0.001$, FWE corrected on the cluster-level at $p < 0.05$.

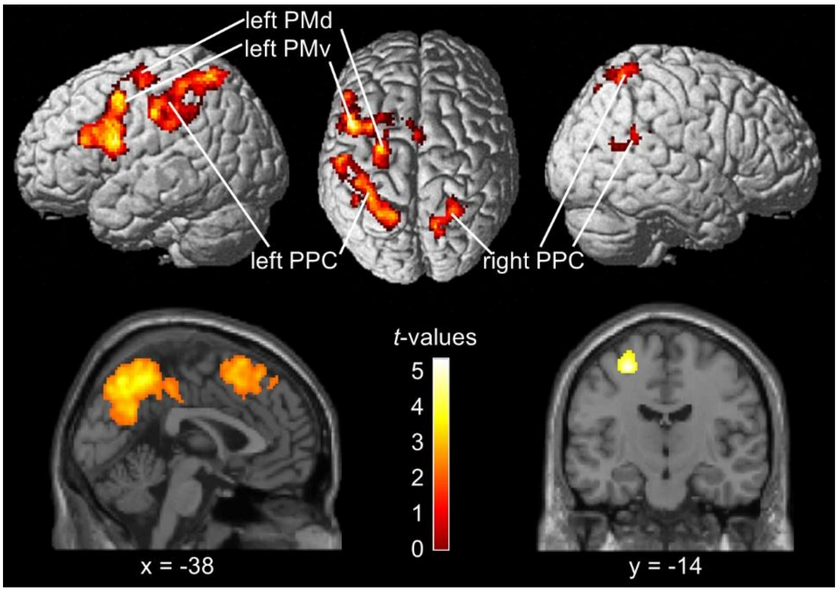

Supplementary Fig. 1. Results of the control analysis which included only a subsample of trials to balance out the amount of left/right motor responses and task rules (comparison of f1 against f2 and comparison of f2 against f1). The analysis revealed above-chance decoding accuracies in the left PMd, the left PMv and the bilateral PPC. Results are displayed at a voxel-level threshold of $p < 0.001$, FWE corrected on the cluster-level at $p < 0.05$. The unthresholded statistical map is accessible via https://neurovault.org/collections/WILTPYNG/images/896901/.

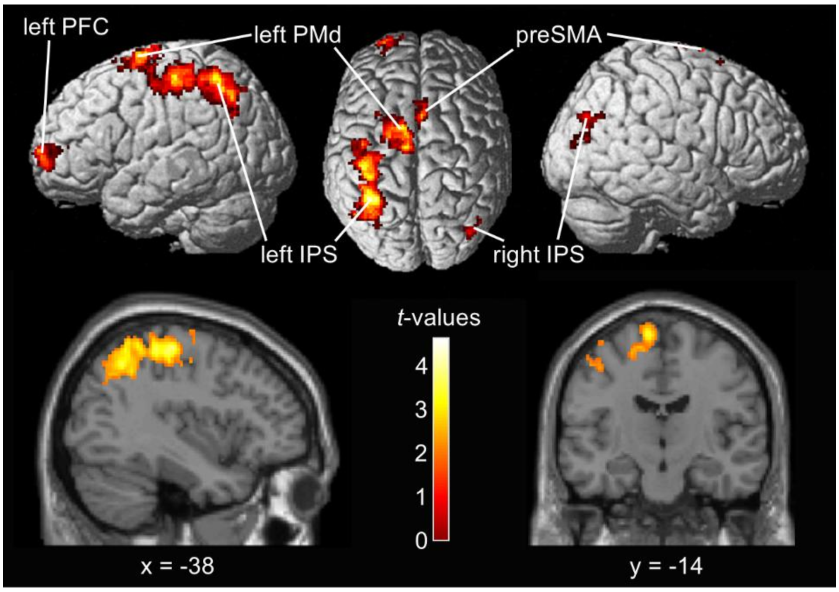

Supplementary Fig. 2. Results of the conjunction analysis, testing against the global null hypothesis (Price & Friston, 1997, Friston et al., 2005). Among other regions, the conjunction confirmed above-chance decoding accuracies in the bilateral IPS and the left PMd. Results are displayed at a voxel-level threshold of $p < 0.001$, FWE corrected on the cluster-level at $p < 0.05$. The unthresholded statistical map is accessible via https://neurovault.org/collections/WILTPYNG/images/896900/

References

Friston, K. J., Penny, W. D., & Glaser, D. E. (2005). Conjunction revisited. NeuroImage, 25(3), 661–667. https://doi.org/10.1016/j.neuroimage.2005.01.013

Price, C. J., & Friston, K. J. (1997). Cognitive conjunction: A new approach to brain activation experiments. NeuroImage, 5(4 Pt 1), 261–270. https://doi.org/10.1006/nimg.1997.0269
